# Cerebral Venous Sinus Thrombosis in the Setting of Hepatic Dysfunction and Viral Hepatitis Seropositivity: A Case Report

**DOI:** 10.7759/cureus.111158

**Published:** 2026-06-19

**Authors:** Maria Nagi, Ursala Adil, Fnu Hikmat Ullah, Ahmad Elsaid, Ibrahim R Raza

**Affiliations:** 1 Internal Medicine, Mayo Hospital, Lahore, PAK; 2 Internal Medicine, Trinitas Regional Medical Center, Elizabeth, USA; 3 Internal Medicine, Ameer-ud-Din Medical College, Lahore, PAK; 4 Internal Medicine, RWJBarnabas Health, West Orange, USA; 5 Internal Medicine, Allama Iqbal Medical College, Lahore, PAK

**Keywords:** cerebral venous sinus thrombosis, hemorrhagic infarction, hepatocellular injury, rebalanced hemostasis, viral hepatitis

## Abstract

Cerebral venous sinus thrombosis (CVST) is an uncommon yet potentially serious cerebrovascular condition characterized by thrombosis of the intracranial venous sinuses. Its presentation is often nonspecific and clinically variable, which may delay diagnosis. Hepatic dysfunction, including hepatocellular injury in the setting of viral hepatitis seropositivity, may be associated with complex hemostatic changes; however, the relationship between hepatic inflammation and venous thrombosis remains difficult to establish in individual cases. We describe the case of a 35-year-old man who presented with acute left-sided pulsatile headache associated with epigastric pain, vomiting, and mild conjunctival discoloration. Laboratory evaluation revealed elevated hepatic transaminases, mild coagulation abnormality, and positive hepatitis B surface antigen and anti-hepatitis C virus antibody serology, suggestive of hepatocellular injury in the setting of viral hepatitis seropositivity. Neuroimaging demonstrated thrombosis involving the left transverse and sigmoid sinuses with associated hemorrhagic infarction affecting the left temporal lobe and cerebellar hemisphere. The patient was managed conservatively with close neurological and hepatic surveillance and experienced progressive symptomatic improvement. This case highlights the importance of considering CVST in patients with persistent or atypical headache, even when neurological examination is initially nonfocal. Although hepatic inflammation and viral hepatitis seropositivity may represent possible contributing factors, the association remains hypothesis-generating, particularly in the absence of confirmatory viral load testing, cryoglobulin testing, and complete thrombophilia evaluation.

## Introduction

Cerebral venous sinus thrombosis (CVST) is an uncommon but potentially life-threatening form of stroke caused by thrombosis of the intracranial venous sinuses, resulting in impaired cerebral venous drainage, venous congestion, and, in some cases, hemorrhagic infarction. It accounts for approximately 0.5% to 1% of all strokes, with reported annual incidence estimates generally ranging from approximately 1.3 to 1.6 cases per 100,000 persons in population-based studies [1]. Its clinical presentation is highly variable, ranging from isolated headache to focal neurological deficits, seizures, or altered consciousness, often leading to delayed diagnosis [1]. Established risk factors include inherited and acquired prothrombotic states, infections, malignancy, dehydration, and systemic inflammatory disorders. Hepatic dysfunction has traditionally been associated with a bleeding tendency due to impaired synthesis of coagulation factors. However, liver disease may involve more complex hemostatic changes than are reflected by routine coagulation tests alone, as alterations in both procoagulant and anticoagulant pathways can create an unstable balance in which either bleeding or thrombosis may occur depending on the clinical context [2,3].

The coexistence of CVST and acute hepatocellular injury in the setting of viral hepatitis seropositivity is rarely reported and presents a significant diagnostic and therapeutic challenge, particularly when routine coagulation tests may suggest impaired hemostasis. In patients with hepatic dysfunction, conventional parameters such as the international normalized ratio (INR) primarily reflect alterations in procoagulant factor synthesis and should not be interpreted as direct markers of thrombotic risk in individuals who are not receiving anticoagulation. Instead, liver disease may produce a rebalanced but unstable hemostatic state, in which reductions in both procoagulant and anticoagulant factors can permit either bleeding or thrombosis depending on the clinical context. This case report describes a patient with hepatic dysfunction and positive hepatitis B and C serology who developed CVST with hemorrhagic infarction. It further emphasizes the importance of maintaining a high index of suspicion for intracranial thrombotic events in patients with hepatic dysfunction who present with persistent or atypical headache despite apparent coagulation abnormalities [3].

## Case presentation

A 35-year-old man presented to the emergency department with a sudden-onset pulsatile headache localized to the left side of the head for one day. The pain was continuous, moderate to severe in intensity, worsened by exposure to light and noise, and partially relieved with paracetamol. He denied visual symptoms, seizures, focal neurological weakness, sensory deficits, or loss of consciousness. In addition, he described a one-week history of gastrointestinal complaints, including epigastric discomfort, abdominal bloating, left flank pain, nausea, recurrent postprandial vomiting, and constipation. There was no history of fever, hematemesis, melena, or similar previous episodes. His medical history was negative for chronic liver disease, prior thromboembolic events, recent immobilization, trauma, or use of anticoagulant or hormonal therapy. He also denied use of over-the-counter supplements, herbal preparations, anabolic agents, or recreational substances. No identifiable high-risk exposures were reported.

At presentation, the patient was awake, fully oriented, and clinically stable. Vital signs revealed a blood pressure of 130/80 mmHg, pulse rate of 74 beats/minute, respiratory rate of 20 breaths/minute, and oxygen saturation of 96% on ambient air. Physical examination demonstrated mild conjunctival icterus and mild dehydration with a coated tongue. Cardiovascular examination revealed normal heart sounds without audible murmurs. Pulmonary examination was unremarkable, with preserved bilateral vesicular breath sounds. The abdomen was soft, non-distended, and non-tender, with normal bowel sounds. Neurological examination did not reveal focal motor or sensory deficits, and cranial nerve function was intact.

Initial laboratory evaluation demonstrated hepatic enzyme derangement, including an alanine aminotransferase level of 408 U/L and alkaline phosphatase level of 189 U/L. Coagulation studies showed mild abnormality, with an INR of 1.26 and an activated partial thromboplastin time of 21.3 seconds. Hematological indices remained within normal limits, including hemoglobin of 14.1 g/dL, total leukocyte count of 8.6 × 10⁹/L, and platelet count of 343 × 10⁹/L. Total bilirubin was measured at 1.02 mg/dL; therefore, the observed conjunctival discoloration was mild and not associated with marked hyperbilirubinemia on initial testing. Hepatitis B surface antigen and anti-hepatitis C virus antibody were positive; however, hepatitis B virus deoxyribonucleic acid (HBV DNA), hepatitis C virus ribonucleic acid (HCV RNA) viral load testing, and cryoglobulin testing were not available during the index admission. The principal laboratory findings are summarized in Table 1.

**Table 1:** Baseline hematological, hepatic, and coagulation laboratory investigations on admission.

Laboratory Parameter	Result	Reference Range
Hemoglobin	14.1 g/dL	13.5-17.5 g/dL
Hematocrit	42%	41-53%
Mean corpuscular volume	82 fL	80-100 fL
Total leukocyte count	8.6 × 10⁹/L	4-11 × 10⁹/L
Platelet count	343 × 10⁹/L	150-400 × 10⁹/L
Erythrocyte sedimentation rate	18 mm/hour	0-15 mm/hour
Total bilirubin	1.02 mg/dL	0.2-1.2 mg/dL
Alanine aminotransferase	408 U/L	7-56 U/L
Alkaline phosphatase	189 U/L	44-147 U/L
Prothrombin time	11.4 seconds	11-13.5 seconds
Activated partial thromboplastin time	21.3 seconds	25-35 seconds
International normalized ratio	1.26	0.8-1.2

Serological evaluation demonstrated positive hepatitis B surface antigen and anti-hepatitis C virus antibody, while dengue serology was negative. Abdominal ultrasonography showed mild inflammatory changes within the liver without focal hepatic lesions, biliary obstruction, or sonographic features suggestive of chronic liver disease. In conjunction with the elevated transaminase levels and viral seropositivity, these findings were suggestive of acute hepatocellular injury.

In view of the severity and atypical nature of the headache, neuroimaging was pursued. Non-contrast computed tomography of the brain demonstrated a hypodense lesion within the left temporal lobe associated with vasogenic edema, localized mass effect, and effacement of adjacent sulci. Further evaluation with magnetic resonance imaging revealed acute dural venous sinus thrombosis involving the left transverse and sigmoid sinuses, accompanied by hemorrhagic infarction involving the left temporal lobe and left cerebellar hemisphere. Associated vasogenic edema with resultant mass effect was also noted (Figure 1). The principal neuroimaging findings are summarized in Table 2.

**Figure 1:**
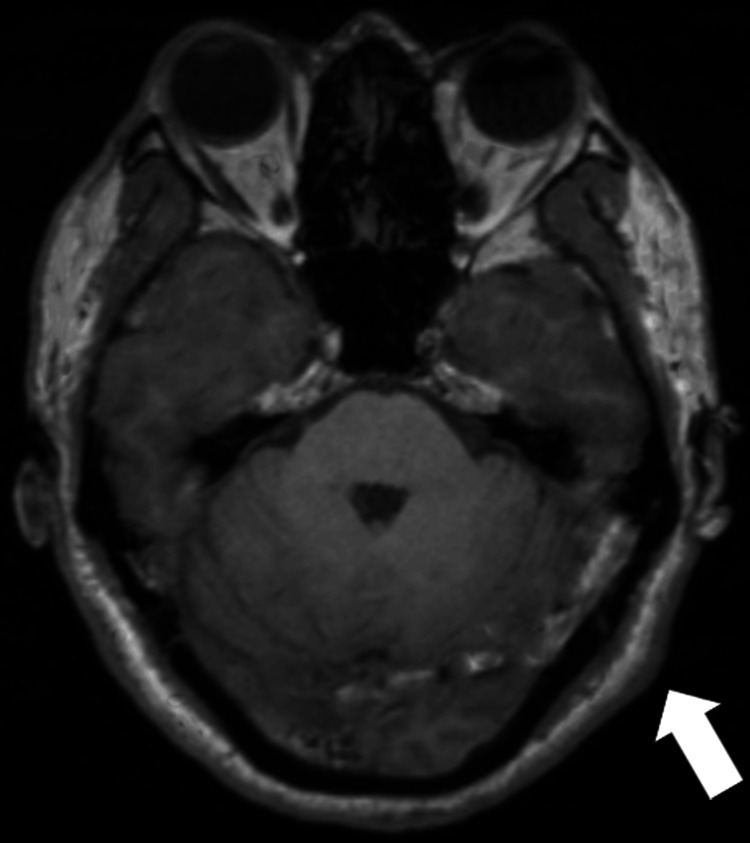
Axial T1-weighted magnetic resonance imaging showing a hyperintense thrombus within the left sigmoid/transverse sinus region, highlighted by the white arrow, consistent with cerebral venous sinus thrombosis.

**Table 2:** Summary of neuroimaging findings in cerebral venous sinus thrombosis.

Modality	Findings
CT brain (non-contrast)	Left temporal lobe hypodensity with vasogenic edema and mass effect
MRI brain	Thrombosis of left transverse and sigmoid sinuses
	Hemorrhagic infarction in left temporal lobe
	Hemorrhagic infarction in left cerebellar hemisphere
	Surrounding vasogenic edema causing mass effect

The patient was admitted for multidisciplinary management involving neurology and gastroenterology teams. He was managed with intravenous fluids for dehydration, antiemetics for symptomatic relief, and close monitoring of liver function and coagulation parameters. Neurological status was closely observed for any signs of raised intracranial pressure or clinical deterioration. In view of the presence of hemorrhagic infarction, the decision regarding anticoagulation was approached cautiously, and a risk-benefit assessment was undertaken by the neurology team, with initial conservative management and close observation during the acute phase. The patient remained hemodynamically stable throughout hospitalization, with preserved consciousness and no development of seizures or focal neurological deficits.

During the hospital course, the patient demonstrated gradual symptomatic improvement, particularly in headache intensity and gastrointestinal complaints. Serial monitoring showed stabilization of liver enzyme levels without progression of coagulation abnormalities. Antiviral therapy for hepatitis B or hepatitis C was not initiated during the index admission, as confirmatory HBV DNA and HCV RNA testing were not available at that time; further viral characterization and treatment planning were deferred to outpatient gastroenterology follow-up. At the time of discharge, the patient was neurologically intact, with significant resolution of symptoms. He was advised regular outpatient follow-up with neurology and gastroenterology services, continued monitoring of liver function and coagulation profile, further evaluation for potential prothrombotic predisposition if clinically indicated, and assessment for hepatitis-directed therapy once confirmatory virological testing became available.

## Discussion

The present case describes CVST occurring in a patient with hepatocellular enzyme elevation and viral hepatitis seropositivity, raising a possible but unproven association between hepatic inflammation and venous thrombosis. This relationship should be interpreted cautiously, as CVST has a broad differential diagnosis and may arise from several well-established etiologies, including inherited or acquired thrombophilia, systemic inflammation, infection, malignancy, dehydration, and other transient prothrombotic states. In this patient, hepatic synthetic function appeared relatively preserved, with normal total bilirubin and prothrombin time (PT) and only mild coagulation abnormality on routine testing. Therefore, liver dysfunction or viral hepatitis seropositivity cannot be considered a definitive or likely sole explanation for CVST. Nevertheless, liver disease and viral hepatitis may, in selected clinical contexts, be associated with hemostatic alterations that are not fully captured by conventional coagulation tests. Because confirmatory HBV DNA testing, HCV RNA testing, cryoglobulin testing, and a complete thrombophilia evaluation were not available during the index admission, the association in this case remains hypothesis-generating rather than causal. This case therefore emphasizes the need to consider CVST in patients with persistent or atypical headache while maintaining a broad diagnostic approach and avoiding overattribution to hepatic abnormalities alone [4].

CVST is an uncommon but potentially life-threatening form of stroke with a highly variable clinical presentation. Headache is the most frequent presenting symptom, yet its nonspecific nature often leads to diagnostic delay, particularly in the absence of focal neurological deficits [5]. In the present patient, the unilateral pulsatile headache resembled a primary headache disorder; however, the persistence and severity of symptoms prompted neuroimaging, which confirmed thrombosis of the left transverse and sigmoid sinuses with associated hemorrhagic infarction. This emphasizes a key clinical principle: the absence of overt neurological deficits does not exclude significant intracranial pathology. In patients with systemic illness, including hepatic dysfunction, new-onset or atypical headache should prompt early consideration of secondary causes such as CVST, as timely imaging is critical for accurate diagnosis and prevention of adverse outcomes.

An additional layer of complexity in this case is the coexistence of hemorrhagic infarction with venous thrombosis. Hemorrhagic transformation is a recognized consequence of CVST, resulting from increased venous pressure, disruption of the blood-brain barrier, and subsequent parenchymal bleeding. Thus, the presence of intracerebral hemorrhage does not exclude an underlying thrombotic process; rather, it may be part of the disease spectrum. In the present case, routine coagulation testing showed only mild abnormality, and hepatic synthetic function appeared relatively preserved. Therefore, the hemorrhagic component should be interpreted primarily in the context of venous infarction rather than as evidence of a systemic bleeding tendency. This underscores the importance of integrating clinical presentation with imaging findings rather than relying solely on routine laboratory values when evaluating complex cases [6,7].

While the coexistence of hepatocellular injury, viral hepatitis seropositivity, and CVST in this patient is clinically noteworthy, it should be interpreted cautiously. The presence of hepatitis B surface antigen and anti-hepatitis C virus antibody, together with elevated transaminase levels, supports hepatocellular injury in the setting of viral hepatitis seropositivity. However, the precise acuity and individual etiological contribution of each viral marker could not be definitively determined. In addition, confirmatory viral load testing, cryoglobulin testing, and a comprehensive evaluation for alternative prothrombotic conditions were not available during the index admission, limiting causal interpretation [8]. Therefore, hepatic dysfunction or viral hepatitis seropositivity cannot be established as the cause of thrombosis in this case. The case remains clinically informative because it reinforces the need to maintain a broad differential diagnosis for CVST and to pursue early neuroimaging in patients with persistent or atypical headache, even when the neurological examination is initially nonfocal.

## Conclusions

This case highlights CVST with hemorrhagic infarction in a patient with hepatocellular enzyme elevation and viral hepatitis seropositivity. Although hepatic inflammation or viral hepatitis seropositivity may represent possible contributing factors, a causal relationship cannot be established in this case, particularly because hepatic synthetic function appeared relatively preserved, and confirmatory viral load testing, cryoglobulin testing, and complete thrombophilia evaluation were not available during the index admission. The key clinical implication is that persistent or atypical headache should not be dismissed, even when the neurological examination is initially nonfocal, and early neuroimaging is essential when CVST is clinically suspected. Overall, this case supports a cautious, broad-differential approach to CVST rather than overattribution of venous thrombosis to hepatic abnormalities alone.
